# Classifying abnormalities in chest radiographs from Vietnam using deep learning for early detection of cardiopulmonary diseases

**DOI:** 10.3389/fradi.2025.1703927

**Published:** 2025-11-20

**Authors:** Chiharu Kai, Satoshi Kasai, Rei Teramoto, Akifumi Yoshida, Hideaki Tamori, Satoshi Kondo, Phan Thanh Hai, Nguyen Van Cong, Dinh Minh Tuan, Thai Van Loc, Naoki Kodama

**Affiliations:** 1 Department of Intelligent Information Engineering, Research Promotion Unit, School of Medical Sciences, Fujita Health University, Toyoake-City, Aichi, Japan; 2 Department of Radiological Technology, Faculty of Medical Technology, Niigata University of Health and Welfare, Niigata-City, Niigata, Japan; 3 Faculty of Radiological Technology, School of Medical Sciences, Fujita Health University, Toyoake-City, Aichi, Japan; 4 The Asahi Shimbun Company, Chuo-ku, Tokyo, Japan; 5 Graduate School of Engineering, Muroran Institute of Technology, Muroran-City, Hokkaido, Japan; 6 Medic Medical Center, Ho Chi Minh-City, Vietnam; 7 MEDICEN Co. Ltd., Ho Chi Minh-City, Vietnam

**Keywords:** chest radiographs, artificial intelligence, vision transformer, infectious diseases, cardiopulmonary diseases

## Abstract

**Introduction:**

Vietnam still faces a high burden of infectious diseases compared with developed countries, and improving its health and sanitation environment is essential for addressing both infectious and non-communicable diseases. Chest radiography is key for early detection of cardiopulmonary diseases. Artificial Intelligence (AI) research on detecting cardiopulmonary diseases from chest radiographs has advanced; however, no AI development studies have used Vietnamese data, despite its high burden of both disease types, for early detection. Therefore, we aimed to develop an AI model to classify normal and abnormal images using a Vietnamese chest radiograph dataset.

**Methods:**

We retrospectively analyzed 12,827 normal and 4,644 abnormal cases from two Vietnamese institutions. Features were derived from principal component analysis and extracted using Vision Transformer and EfficientnetV2. We performed binary classification of normal and abnormal images using Light Gradient Boosting Machine with 5-fold cross-validation.

**Results:**

The model achieved an F1-score of 0.668, sensitivity of 0.596, specificity of 0.931, accuracy of 0.842, and AUC of 0.897. Subgroup evaluation revealed high accuracy in both infectious and non-communicable cases, as well as in urgent cases.

**Conclusion:**

We developed an AI system that classifies normal and abnormal chest radiographs with high clinical accuracy using Vietnamese data.

## 1 Introduction

In recent years, Vietnam has seen an increase in deaths due to non-communicable diseases. However, compared with developed countries, the burden of infectious diseases remains high, highlighting the need to improve health and sanitation from the perspective of both infectious and non-communicable diseases (1). In 2021, ischemic heart disease, COVID-19, chronic obstructive pulmonary disease, lung cancer, and tuberculosis were among the top 10 causes of death in Vietnam, with cardiopulmonary diseases accounting for half of all deaths, indicating that early detection of these conditions is a critical public health priority (2). This issue extends across Asia; in Southeast Asia, East Asia, and Oceania, ischemic heart disease, chronic obstructive pulmonary disease, and tracheal, bronchial, and lung cancer ranked as the second to fourth leading causes of death in 2021 (2).

Chest radiographs are the most frequently performed initial imaging examination for patients with cardiopulmonary symptoms, because of their wide availability, cost-effectiveness, ability to detect a broad range of conditions, and low radiation dose (3). For example, in prevalence surveys across 33 African and Asian countries, 30%–79% of individuals with microbiologically confirmed tuberculosis were asymptomatic and detected only through chest radiography (4–6). Given this, early detection of cardiopulmonary diseases via chest radiographs, regardless of whether they are infectious or non-communicable, could enable earlier intervention and improve patient outcomes. In Southeast and South Asia, including Vietnam, many health centers lack advanced imaging equipment such as computed tomography, making chest radiography the only available imaging modality. Therefore, chest radiography is particularly important in such regions.

Given the importance of chest radiographs in early diagnosis, artificial intelligence (AI) research to detect cardiopulmonary diseases from these images has been actively conducted (7–24). However, most studies have focused on AI models for specific diseases, such as cardiac disease (7), pneumothorax (8), lung cancer (9, 10), tuberculosis (11–14), pneumonia (15), COVID-19 (16), and pneumoconiosis (17). Annalise.ai, for instance, can detect 127 clinical findings from chest radiographs but does not provide disease diagnoses (24). Some studies have developed AI to classify chest radiographs as normal or abnormal rather than identifying specific diseases (25–30). However, these often lack sufficient representation of infectious or cardiovascular diseases (25–28) or use datasets with more abnormal than normal cases, creating imbalanced distributions that do not reflect real-world clinical reality (29, 30). To date, no AI-development studies have been conducted using Vietnamese data, despite the country's unique dual burden of infectious and non-communicable diseases, for the early detection of cardiopulmonary abnormalities.

Therefore, this study aimed to develop an AI model to classify normal and abnormal chest radiographs using a dataset of Vietnamese patient images with a high representation of both infectious and non-communicable diseases.

## 2 Materials and methods

### 2.1 Study design

Chest radiographs were collected retrospectively from two Vietnamese institutions: Medic Medical Center and MEDICEN Co., Ltd., both located in Ho Chi Minh City, Vietnam. The study was conducted in accordance with the Declaration of Helsinki and approved by the Institutional Review Board of Niigata University of Health and Welfare (approval number: 19648-250819). This manuscript adheres to the Standards for Reporting of Diagnostic Accuracy Studies guidelines (31).

### 2.2 Dataset

**Figure 1:**
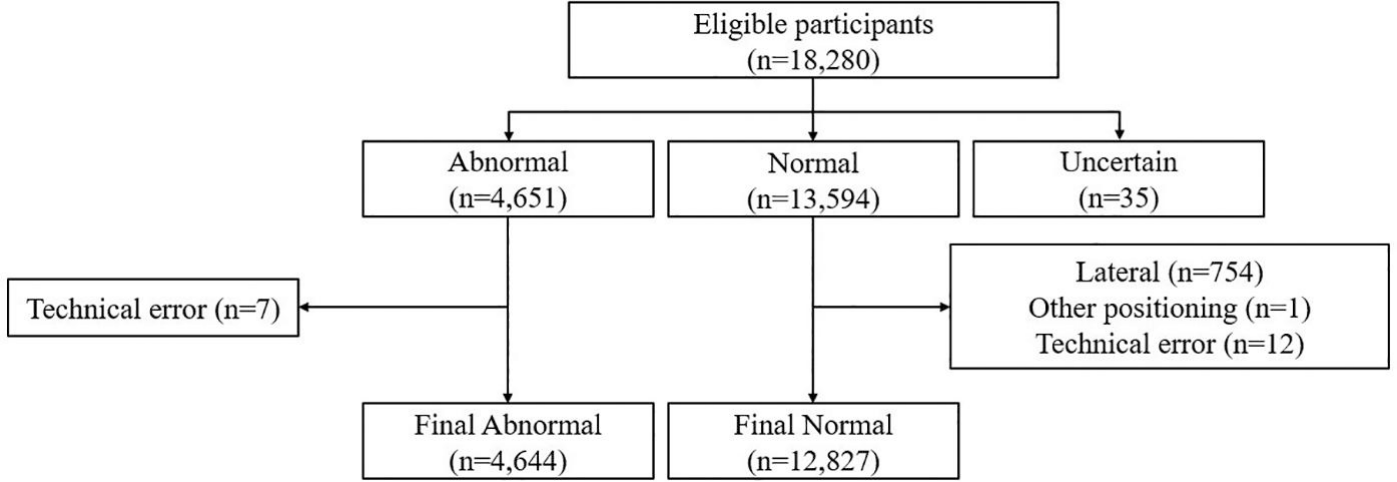
Flow diagram of the eligibility criteria for the dataset used in this study (*n* = total cases).

Data were collected at Medic Medical Center from individuals aged 18 years and older between January 1 and December 31, 2024. MEDICEN Co., Ltd. collected data from the same age group during two periods—July 2 to October 11, 2021, and May 6 to August 19, 2024. In this study, data were collected using an opt-out procedure. Patients were provided easy access to explanatory materials and given the opportunity to decline participation. A total of 18,280 cases with linked imaging and clinical information were included. Personal identifiers such as patient name, age, and sex were removed. Personal information within the images were obscured using black-box masking. Based on clinical annotations, cases were categorized as normal, abnormal, or uncertain. These determinations were made by two radiologists with 35 and 8 years of experience, based on diagnostic imaging reports generated in routine practice. Only frontal chest radiographs were included in the final dataset. Figure 1 illustrates the eligibility criteria for the datasets used in this study. In the normal category, 754 cases were excluded owing to lateral chest radiographs, one case had images of non-thoracic body parts, and 12 cases had technical errors. In the abnormal category, seven cases with technical errors were excluded.

### 2.3 Pre-processing

The chest radiographs were converted from DICOM images to PNG format. Pixel values were linearly scaled and normalized to 8-bit depth such that the top 1% of intensities were set to 255 and the bottom 7% to 100, adjustments made while subjectively evaluating the contrast of the pre-processing images to account for contrast changes caused by black-box masking. Image sizes were resized to 224 × 224 pixels using padding and resizing while preserving aspect ratio. For images with MONOCHROME1 photometric interpretation, pixel inversion was applied.

### 2.4 Model development

**Figure 2:**
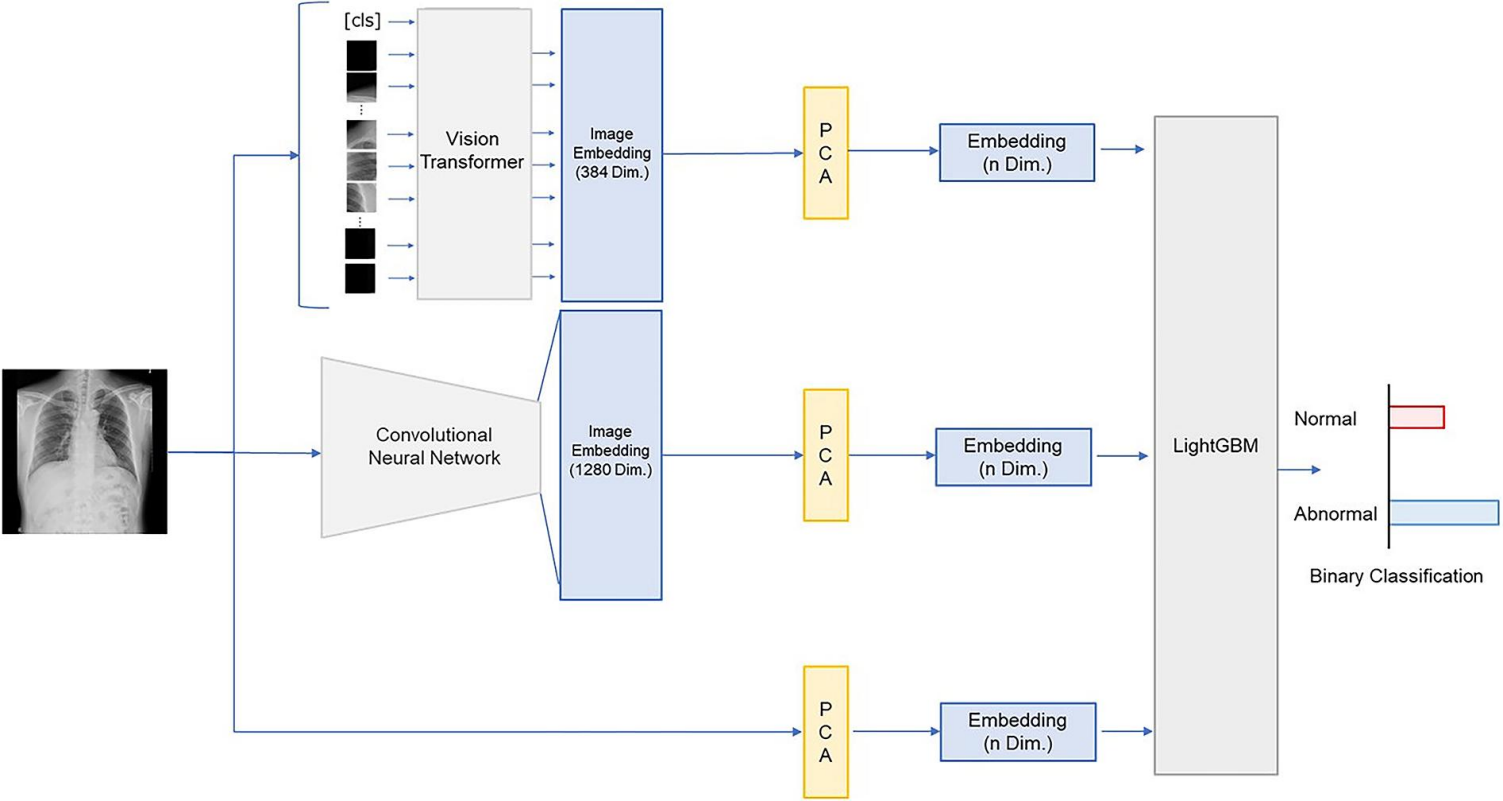
A schematic diagram of the model used in this study. PCA, Principal Component Analysis; LightGBM, Light Gradient Boosting Machine.

A schematic diagram of the model used in this study is shown in Figure 2. A pretrained Vision Transformer (ViT) model [vit_small_patch8_224. dino (32, 33)] from the PyTorch Image Models (timm) library (34) was used to extract 384 features from the pre-processed images. These features were dimensionally compressed using principal component analysis (ViT features). Similarly, a pre-trained convolutional neural network model [tf_efficientnetv2_m.in21k_ft_in1k (35, 36)], also from timm library (34), was used to extract 1,280 features, which were dimensionally compressed via principal component analysis (convolutional neural network features). Additionally, principal component analysis was directly applied to the pre-processed images to extract raw image features. The numbers of dimensional compression and the image features were varied at 4, 8, 16, 32, 64, 128, and 256.

Cases were randomly divided into five folds. Using 5-fold cross-validation, models were trained and hyperparameters tuned. Using ViT, convolutional neural network, and image features from principal component analysis as inputs, Optuna (37) (version 4.3.0) was used to optimize the parameters for the two classifications (normal or abnormal) using a Light Gradient Boosting Machine. The parameter with the highest F1-score for positive (abnormal) cases was adopted. Optimized Light Gradient Boosting Machine parameters included setting the data number and depth for the tree model, feature selection method, learning rate, and L1/2 regularization. Training was set for up to 5,000 iterations with early stopping after 10 rounds. A total of 2,000 parameter trials were performed. Feature importance was assessed using the “split” type, which counts how often a feature is used in splits across the model. All computations were performed on a machine equipped with an 11th Gen Intel(R) Core™ i9-11900K CPU, 64 GB RAM, and an NVIDIA GeForce RTX 3090 GPU, using Python (v3.11.5) and the PyTorch framework (version 2.1.0).

### 2.5 Evaluation methods

The metrics used to evaluate the performance of the binary classification (normal vs. abnormal) were the F1-score, sensitivity, specificity, accuracy, and area under the curve (AUC). These were also evaluated at fixed sensitivity levels of 0.95 and 0.90 and at specificity levels of 0.95 and 0.90. All chest radiographs labeled as abnormal were analyzed for the presence of each of 18 predefined diseases, and the true positive fraction was calculated. The 18 conditions included: atelectasis, chronic obstructive pulmonary disease, infectious pulmonary disease, interstitial pathology, lung tumors, pleural effusion, pneumonia, pneumothorax, pulmonary emphysema, tuberculosis, cardiovascular disease, bronchiectasis, mediastinal tumor, fracture, skeletal muscle abnormalities, flail chest, old scar, and other. In further subgroup analysis the true positive fraction was evaluated based on the number of lesions identified by physicians, and the types of diseases in a single image.

## 3 Results

The final dataset comprised 12,827 normal and 4,644 abnormal cases. The detailed case distribution is presented in Table 1. The total number of diagnosed diseases exceeded the number of abnormal cases, reflecting the presence of multiple diseases per image. Additional details on the dates of chest radiographic examinations and the x-ray systems used are provided in Supplementary File S1.

**Table 1:** Case distribution in the dataset by institution (Medic Medical Center, Medic; MEDICEN. Co. Ltd., Medicen) (*n* = total cases).

		Institution		
		Medic	Medicen	All
Truth	All	14,550	2,921	17,471
	Normal	10,719	2,108	12,827
	Abnormal	3,831	813	4,644
Diseases	All	4,355	950	5,305
	Tuberculosis	422	65	487
	Pneumonia	58	422	480
	Interstitial pathology	1	155	156
	Pleural effusion	86	24	110
	Lung tumors	16	12	28
	Chronic obstructive pulmonary disease	20	5	25
	Infectious pulmonary disease	0	24	24
	Atelectasis	6	12	18
	Bronchiectasis	1	5	6
	Pneumothorax	2	3	5
	Pulmonary emphysema	0	2	2
	Cardiovascular disease	3,134	52	3,186
	Fracture	311	15	326
	Old scar	162	69	231
	Other	123	76	199
	Mediastinal tumor	9	4	13
	Abnormalities of skeletal muscles	0	5	5
	Flail chest	4	0	4

The highest classification was achieved when the number of principal component analysis dimensions was set to 256, yielding an F1-score of 0.668 (95% CI: 0.656–0.681), sensitivity of 0.596 (95% CI: 0.582–0.610), specificity of 0.931 (95% CI: 0.927–0.936), accuracy of 0.842 (95% CI: 0.837–0.848), and AUC of 0.897 (95% CI: 0.892–0.902). Results of the 5-fold cross-validation, along with performance at fixed sensitivity (0.90 and 0.95) and specificity (0.90 and 0.95), are shown in Table 2.

**Table 2:** Results of 5-fold cross-validation and fixed sensitivity (0.95 and 0.90) and specificity (0.95 and 0.90).

All	F1-score	Sensitivity	Specificity	Accuracy	AUC
	0.668	0.596 (2,770/4,644)	0.931 (11,948/12,827)	0.842 (14,718/17,471)	0.897
Fold 1	0.657	0.588 (546/929)	0.927 (2,379/2,566)	0.837 (2,925/3,495)	0.895
Fold 2	0.682	0.612 (569/929)	0.933 (2,394/2,565)	0.848 (2,963/3,494)	0.902
Fold 3	0.662	0.591 (549/929)	0.929 (2,384/2,565)	0.839 (2,933/3,494)	0.894
Fold 4	0.676	0.598 (556/929)	0.938 (2,406/2,565)	0.848 (2,962/3,494)	0.901
Fold 5	0.663	0.593 (550/928)	0.929 (2,385/2,566)	0.840 (2,935/3,494)	0.895
Sensitivity 0.95	0.626	0.950 (4,413/4,644)	0.607 (7,781/12,827)	0.698 (12,194/17,471)	–
Sensitivity 0.90	0.673	0.900 (4,179/4,644)	0.719 (9,223/12,827)	0.767 (13,402/17,471)	–
Specificity 0.90	0.702	0.691 (3,208/4,644)	0.900 (11,545/12,827)	0.844 (14,753/17,471)	–
Specificity 0.95	0.623	0.515 (2,392/4,644)	0.950 (12,186/12,827)	0.834 (14,578/17,471)	–

AUC, Area under the curve.

Table 3 presents the true positive fraction for each of the 18 diseases in the abnormal cases, corresponding to the conditions shown in Table 2; five-fold cross-validation, fixed sensitivity (0.90 and 0.95), and fixed specificity (0.90 and 0.95). Table 4 shows the true positive fraction stratified by the number of lesions identified by physicians, and the types of diseases in a single image.

**Table 3:** True positive fraction for each of the 18 diseases in abnormal cases.

Category	Diseases	Number of diseases	5-fold cross-validation	Sensitivity 0.95	Sensitivity 0.90	Specificity 0.90	Specificity 0.95
Pulmonary diseases	Tuberculosis	487	0.419	0.875	0.801	0.542	0.343
	Pneumonia	480	0.804	0.985	0.977	0.885	0.752
	Interstitial pathology	156	0.891	1.000	0.994	0.923	0.846
	Pleural effusion	110	0.500	0.927	0.827	0.591	0.473
	Lung tumors	28	0.500	0.964	0.893	0.571	0.357
	Chronic obstructive pulmonary disease	25	0.560	0.960	0.840	0.760	0.440
	Infectious pulmonary disease	24	0.917	0.958	0.958	0.917	0.833
	Atelectasis	18	0.944	1.000	1.000	0.944	0.889
	Bronchiectasis	6	0.833	1.000	1.000	1.000	0.833
	Pneumothorax	5	0.800	1.000	1.000	0.800	0.800
	Pulmonary emphysema	2	0.500	1.000	1.000	0.500	0.500
Non-pulmonary diseases	Cardiovascular disease	3,186	0.616	0.975	0.927	0.712	0.532
	Fracture	326	0.567	0.923	0.880	0.675	0.466
	Old scar	231	0.563	0.913	0.866	0.649	0.459
	Other	199	0.397	0.799	0.714	0.487	0.347
	Mediastinal tumor	13	0.538	1.000	0.923	0.769	0.462
	Abnormalities of the skeletal muscles	5	0.600	0.600	0.600	0.600	0.600
	Flail chest	4	0.250	1.000	0.750	0.750	0.250

**Table 4:** True positive fraction by the number of lesions identified by physicians, and the types of diseases in a single image.

Stratification		Number of cases	Number of true positives	True positive fraction
Number of lesions	1	3,505	1,982	0.565
	2	843	576	0.683
	3	197	138	0.701
	4	56	41	0.732
	5	24	18	0.750
	6	12	10	0.833
	7	4	2	0.500
	8	2	2	1.000
	13	1	1	1.000
Type of diseases	1	4,036	2,351	0.583
	2	561	384	0.684
	3	43	31	0.721
	4	4	4	1.000

## 4 Discussion

Using a dataset of Vietnamese chest radiographs with a high representation of both infectious and non-communicable diseases, we developed an AI model to classify normal and abnormal cases, achieving an F1-score of 0.668, sensitivity of 0.596, specificity of 0.931, accuracy of 0.842, and AUC of 0.897. Nguyen et al. developed an AI system trained on non-Vietnamese data and reported an F1-score of 0.653 and accuracy of 0.796 when evaluated on Vietnamese datasets (38). Our model demonstrated higher performance, suggesting strong potential for clinical application in Vietnam. Furthermore, when compared with previous studies from India (South Asia), a different region from Vietnam but with a similar disease spectrum, Nabulsi et al. developed an AI system trained on Indian data and reported a sensitivity of 0.63, specificity of 0.91, and AUC of 0.87 on Indian test sets (27). Govindarajan et al. evaluated the commercially available AI algorithm qXR (Qure.ai Technologies, Mumbai, India) using Indian data and reported a sensitivity of 0.879, specificity of 0.829, and AUC of 0.871 (39). In our study, the AUC was 0.897; sensitivity was 0.691 at a fixed specificity of 0.900, and specificity was 0.719 at a fixed sensitivity of 0.900. These results are comparable to, or exceed, those reported in AI development studies and commercial AI systems evaluated in the South Asian region. The F1-score of 0.668 in this study also surpasses the average F1-score of 0.387 achieved by four radiologists in a pneumonia detection task using randomly selected chest radiographs from Chestx-ray14 dataset (40). Furthermore, in the binary classification of normal versus abnormal radiographs (486 normal, 529 abnormal), our model's performance was comparable with that of five non-radiologists (sensitivity, 0.699; specificity, 0.901; AUC, 0.814), although it was inferior to that of board-certified radiologists and thoracic radiologists (26).

The high accuracy for infectious diseases (true positive fraction of 0.917 for infectious pulmonary disease and 0.885 for pneumonia, at specificity of 0.90) makes the system valuable in Vietnam, where infectious diseases are common. For non-communicable diseases, the model achieved high accuracy for conditions with broad radiographic manifestations, such as interstitial pathology, pneumothorax, and atelectasis (true positive fraction: 0.923, 0.800, and 0.944, respectively, at specificity of 0.90). High accuracy for urgent diseases such as pneumothorax (true positive fraction 0.800) and mediastinal tumor (true positive fraction 0.769) at specificity of 0.90 further enhances its clinical utility.

Regarding the false-negative cases for cardiovascular disease, which accounted for the greatest number of cases and for pneumothorax, an urgent condition, we found that the lung fields were symmetrically delineated and closely resembled those of normal cases. Because these cases are very easy to misinterpret with the naked eye, distinguishing normal from abnormal is difficult and may result in false negatives. AI showed a tendency toward higher true positive fraction with increasing number and diversity. This capability could help prioritize complex or critical cases, offering high clinical value. This research is based on the premise that the technology will eventually be incorporated into workflow as a triage tool, for example, in chest-radiograph screening. In computer-aided diagnosis (CAD) research, we refer to this as computer-aided triage (CADt), which directs patients with possible abnormal lesions to immediate physician interpretation. Consequently, chest radiographs obtained during screening are fed to the AI system immediately after acquisition. When an abnormality is detected, the physician's diagnosis and intervention can be prioritized right away. In CADt, it is important to select cases that have a high probability of requiring priority over routine readings. Our system can forward 90 % of prevalent cases for priority review while preserving a high true-positive fraction for each disease and achieving a very high negative predictive value of 0.952 (sensitivity = 0.900). Therefore, we believe it has strong potential for effective clinical use, although further improvements in sensitivity are needed.

Southeast Asia faces a significant shortage of medical personnel, often associated with long working hours and low wages. This challenge has been exacerbated by the COVID-19 pandemic. In many cases, patients experience long wait times or receive no consultation because of a shortage of medical staff, particularly in rural areas. Vietnam faces this same issue. Despite rapid economic growth, the country must urgently address its healthcare workforce shortage; as life expectancy rises, the aging population expands and demand for medical services increase (41, 42). The AI system developed in this study, trained on Vietnamese data, demonstrated high accuracy for triaging infectious and urgent diseases and for identifying cases with multiple pathologies. Its diagnostic performance, which was comparable to that of non-radiology physicians, suggests its potential to significantly improve the workflow of medical staff, even if it does not yet match the accuracy of radiologists. Therefore, this system could be a valuable tool not only in Vietnam but also in other Southeast Asian countries, such as the Philippines and Cambodia (1, 42), which face similar disease patterns and shortages of medical personnel. This is expected to enhance healthcare delivery in the region.

We developed an AI model to classify images as normal or abnormal, with subsequent analysis of its true positive fraction for 18 specific abnormalities. Disease-specific analyses were conducted to develop a system for triaging infectious and urgent diseases using chest radiography. In this study, we focused on the importance of analyzing data on infectious and urgent diseases rather than on the findings. However, accuracy was moderate for typical infectious diseases, such as tuberculosis, and diseases with localized manifestations, such as lung tumors. As data collection expands, improving the model's performance across all disease types will be an important goal for future development.

Chest radiographs and clinical information used in this study were obtained from two institutions in Vietnam. To implement this system in clinical practice, the AI model must be optimized, and a multicenter prospective study involving additional imaging systems, populations, and institutions should be conducted.

In conclusion, we developed an AI model capable of classifying normal and abnormal chest radiographs with performance comparable with that of non-radiologist physicians, using a Vietnamese dataset rich in both infectious and non-communicable diseases. This system has the potential to improve the prognosis of patients in Vietnam, where there is a shortage of medical staff, by enabling the early detection of cardiopulmonary diseases regardless of whether they are infectious or non-communicable, thereby allowing for timely intervention.

## Data Availability

The datasets presented in this article are not readily available because they contain information that could compromise the privacy of research participants. Requests to access the datasets should be directed to Naoki Kodama, kodama@nuhw.ac.jp.
