# Supplementary material for: Classifying abnormalities in chest radiographs from Vietnam using deep learning for early detection of cardiopulmonary diseases
Source: Front Radiol. 2025 Nov 20;5:1703927. doi: 10.3389/fradi.2025.1703927 (PMC12675158; doi:10.3389/fradi.2025.1703927)
Supplement: Supplementary file 1 [file Table1.docx]

Supplementary Material

# Supplementary Table 1

## Details on the dates of chest radiographic examinations and the X-ray systems used in the dataset Supplementary Figures

| Modality, X-ray system, Country | Normal (n = total cases) | | Abnormal (n = total cases) | |
| --- | --- | --- | --- | --- |
|  | Medic | Medicen | Medic | Medicen |
|  | January 1 to December 28, 2024 | July 2 to October 11, 2021, and  May 6 to August 15, 2024 | January 1 to December 31, 2024 | July 2 to October 13, 2021, and  May 6 to August 19, 2024 |
|  | 10,719 | 2,108 | 3,831 | 813 |
| DX, KONICA MINOLTA, INC., Japan | 7,682 | 0 | 3,168 | 0 |
| CR, CANON MEDICAL SYSTEMS CORPORATION, Japan | 0 | 0 | 9 | 0 |
| DX, Samsung Medison Co., Ltd., Korea | 2,170 | 0 | 642 | 0 |
| CR, DRTECH Co., Ltd., Korea | 867 | 0 | 11 | 0 |
| DX, Vieworks Co., Ltd., Korea | 0 | 57 | 0 | 268 |
| CR, uncertain | 0 | 0 | 1 | 0 |
| DR, uncertain | 0 | 81 | 0 | 323 |
| DX, uncertain | 0 | 1,970 | 0 | 222 |

Abbreviations: Medic Medical Center: Medic; MEDICEN. Co., Ltd.: Medicen; DX: Digital X-ray; CR: Computed Radiography; DR: Digital Radiography
